# Anti-Inflammatory Activity of *Delonix regia* (Boj. Ex. Hook)

**DOI:** 10.1155/2012/789713

**Published:** 2011-11-01

**Authors:** Vaishali D. Shewale, Tushar A. Deshmukh, Liladhar S. Patil, Vijay R. Patil

**Affiliations:** ^1^Tapi Valley Education Society, Hon'ble, Loksevak Madhukarrao Chaudhari College of Pharmacy, Faizpur 425 503, India; ^2^Department of Pharmacognosy, Hon'ble, Loksevak Madhukarrao Chaudhari College of Pharmacy, Faizpur 425 503, Maharashtra, India; ^3^Department of Biotechnology, Hon'ble, Loksevak Madhukarrao Chaudhari College of Pharmacy, Faizpur 425 503, Maharashtra, India

## Abstract

The present work was to evaluate the anti-inflammatory activity of *Delonix regia* leaves (Family: Caesalpiniaceae). The powder of *Delonix regia* leaves was subjected to extraction with ethanol in soxhlet extractor. The ethanol extract after preliminary phytochemical investigation showed the presence of sterols, triterpenoids, phenolic compounds and flavonoids. The anti-inflammatory activity was studied using carrageenan-induced rat paw edema and cotton pellet granuloma at a three different doses (100, 200, and 400 mg/kg b.w. p.o.) of ethanol extract. The ethanol extract of *Delonix regia* leaves was exhibited significant anti-inflammatory activity at the dose of 400 mg/kg in both models when compared with control group. Indomethacin (10 mg/kg b.w. p.o) was also shown significant anti-inflammatory activity in both models.

## 1. Introduction

Inflammation is a series of pathological changes associated with local vascular reaction and cellular response, the living tissue, an injury insufficient to kill the tissue. This is distinguished from the wider problem of generalized reactions of the body. However, it is related to infection caused by microorganisms, and various pathological changes are associated with it [1]. Traditional medicines play an important role in health services around the globe. About three-quarters of the world population relies on plants and plant extracts for healthcare. The rational design of novel drugs from traditional medicine offers new prospects in modern healthcare. *Delonix regia *(Boj. Ex. Hook) (Family: Caesalpiniaceae) is a medium-sized tree found in greater parts of India. The decoction of the leaves is traditionally used in treating gastric problems, body pain, and rheumatic pains of joints [2, 3]. Ethanolic extracts of flower and bark were investigated to anti-inflammatory activity in rats [4]. The leaves are reported to antibacterial [5] and antimalarial [6]. *Delonix regia* contains proteins, flavonoids, tannins, phenolic compounds, glycosides, sterols, and triterpenoids. However, no data were found regarding the pharmacological and phytochemical evaluation of the leaves of the plant. The aim of the present study was to investigate the anti-inflammatory activity of the ethanol extract of the leaves of *Delonix regia* (Boj. Ex. Hook).

## 2. Materials and Methods

### 2.1. Plant Material

*Delonix regia* leaves were procured from the local market in Jalgaon, Maharashtra, India, identified, and authenticated. A voucher specimen (voucher specimen number-Vaishu 9200) was deposited in the herbarium of the Department of Botany, Rashtrasant Tukdoji Maharaj, Nagpur University, Nagpur.

### 2.2. Extraction of Plant Material

The leaves were dried under a shade and pulverized. The coarse powder (1000 g) was extracted with ethanol using a soxhlet apparatus. The extract was dried using a rotary vacuum evaporator and stored in a desiccator until further use. The percentage yield of ethanol extract of *Delonix regia* (EEDR) was 25.0% w/w.

### 2.3. Phytochemical Screening

A preliminary phytochemical screening of *Delonix regia *was carried out [7]. The presence of alkaloid (Dragendorff reagent and Mayer's reagent), flavonoids (Shinoda test), steroids (Liberman Burchard test), and terpenes (Vanillin sulfuric acid reagent) were analyzed.

### 2.4. Drugs and Chemicals

Indomethacin was purchased from Themis Pharmaceutical Ltd. India. Carrageenan was purchased from Sigma Aldrich, USA. Tween 80 and other reagents of analytical grade were purchased from S. D. Fine Chem. Ltd, India. 

### 2.5. Animals

Wistar albino rats (150–200 g) and mice (20–25 g) of either sex were purchased from Calcutta Fish Aquarium, Indore, India and were housed under standard conditions of temperature and light. Animals had free access to food (Amrut Feeds, Pune, India) and water. The Institutional Animal Ethics Committee approved the protocol of the study.

### 2.6. Acute Toxicity Studies

Healthy adult Swiss albino mice of either sex weighing between 20 and 25 g were subjected to acute toxicity studies as per guidelines (AOT no. 425) suggested by the Organization for Economic Cooperation and Development [8]. Groups of six mice each were administered orally graded doses ranging from 0.1 to 5 g/kg. The mice were observed continuously for 2 h for behavioral, neurological, and autonomic profiles for any lethality or death for the next 48 h.

### 2.7. Anti-Inflammatory Activity

#### 2.7.1. Carrageenan-Induced Paw Edema

The anti-inflammatory activity of the extract was carried out using Wistar albino rats (150–200 g) of either sex [9, 10]. The rats were divided into five groups of six rats each. The control group received 1% (v/v) Tween 80 in water, p.o. at a dose of 10 mL/kg. The positive control group was treated orally with the standard drug, indomethacin (10 mg/kg). Ethanol extract was administered orally to the other groups in doses of 100, 200, and 400 mg/kg as shown in Table 1. All the suspensions were administered 30 min before the induction of oedema by administering 0.1 mL of 1% w/v carrageenan in saline [11, 12]. The degree of paw oedema of all the groups was measured using a plethysmometer at 0, 1, 3, 5, and 7 h after the administration of carrageenan to each group.

### 2.8. Cotton Pellet Granuloma

Two autoclaved cotton pellets weighing 10 ± 1 mg were implanted subcutaneously into both sides of the groin region of each rat. The rats were divided into five groups of six rats each. The control group received 1% (v/v) Tween 80 in water, p.o. at a dose of 10 mL/kg. The positive control group was treated orally with the standard drug, indomethacin (10 mg/kg). Ethanol extract was administered to the other groups in doses of 100, 200, and 400 mg/kg orally for 7 days. After 7 days, the animals were sacrificed, and the pellets together with the granuloma tissues were carefully removed, dried in an oven at 60°C, weighed, and compared with control [13].

### 2.9. Statistical Analysis

All values are expressed as Mean ± SEM. Statistical analysis was performed by one-way analysis of Variance (ANOVA), and individual comparisons of the group mean values were done using Dunnet's *t*-test, with the help of Graph Pad prism 4.0 software. The value of *P* lower than 0.05 was considered as significant (*P* is probability) [14, 15].

## 3. Results

### 3.1. Phytochemical Studies

The preliminary phytochemical studies of ethanol extract of *Delonix regia* was indicated the presence of sterols, triterpenoids, phenolic compounds, and flavonoids.

### 3.2. Acute Toxicity Studies

In acute oral toxicity study, mice given graded doses ranging from 0.1 to 5 g/kg appeared normal. *Delonix regia* was safe up to a dose level of 5000 mg/kg of body weight. No lethality or any toxic reactions were found up to the end of the study period.

### 3.3. Anti-inflammatory Activity

#### 3.3.1. Carrageenan-Induced Paw Edema

The ethanol extract (400 mg/kg) significantly inhibited carrageenan-induced paw oedema. The ethanol extract produced a dose-dependent inhibition of carrageenan oedema which was comparable with known anti-inflammatory drugs. The ethanol extract of *Delonix regia* produced significant (*P* < 0.01) anti-inflammatory activity. Significant reduction of paw oedema was observed at 3 h after carrageenan injection. The reduction in carrageenan-induced paw oedema by 400 mg/kg of ethanol extract after 3 h was 48.1%, while oedema reduction by the standard drug, indomethacin (10 mg/kg), was 65.8% (Table 1).

#### 3.3.2. Cotton Pellet Granuloma

The ethanol extract significantly inhibited cotton pellet granuloma. The percent inhibition of ethanol extract was 42.4% at dose of 400 mg/kg, and this inhibition was less than that produced by indomethacin (61.6%) (Table 2).

## 4. Discussion

Carrageenan-induced oedema of rat foot is used widely as a working model of inflammation in the search for new anti-inflammatory agents [16] and appeared to be the basis for the discovery of indomethacin, the anti-inflammatory drug [17]. The oedema which develops in rat paw after carrageenan injection is a biphasic event. The initial phase is attributed to the release of histamine and serotonin, the oedema maintained between the first and second phase to kinin, and the second phase to prostaglandin [18]. All the mediators appear to be dependent upon an intact complement system for their activation and release [19]. It has been shown that, in the early phase of the oedema, the dominant cells are polymorphonuclears whereas in advanced stages mononuclears predominate. In this study, indomethacin (nonsteroidal) anti-inflammatory drug was tested on carrageenan oedema [20]. Phytochemical studies on *Delonix regia* revealed that it contains *β*-sitosterol, tannin, lupeol, and flavonoids [21]. Hentriacontane, hentriacontanol and it's D-glucoside, and campesterol were identified as constituents of *Delonix regia *[22]. 

Most of the anti-inflammatory triterpenes isolated have lupane, oleanane, ursane, and taraxastane. Lupeol and ursolic acid showed significant anti-inflammatory activity in various models [23, 24]. Lupeol has been reported to possess dose-dependent suppression of PGE_2_ without any effect of LTC_4_ release. Thus, ursolic acid and lupeol [25] were able to prevent the production of some inflammatory mediators which likely contributed to anti-inflammatory effect of *Delonix regia*. Jiang and Dusting have reported that phenolic compounds have potential role in inflammatory conditions [26]. Satya Prasad et al. have further shown that phenolics inhibit polymorphonuclear lipoxygenase, an enzyme involved in inflammatory conditions [27]. The cotton pellet granuloma method has been widely employed to assess the transductive, exudative, and proliferative components of chronic inflammation and is a typical feature of established chronic inflammatory reaction. The fluid absorbed by the pellet greatly influences the wet weight of the granuloma, and dry weight correlates well with the granuloma of the granulomatous tissue formed [28, 29]. Administration of ethanol extract at the doses of 400 mg/kg significantly reduced the granulomatous tissue formation when compared to control.

## 5. Conclusion

Preliminary phytochemical analysis indicated that the ethanol extract of *Delonix regia *contains sterols, triterpenoids, flavonoids, and phenolic compounds. The anti-inflammatory activity of ethanol extract of *Delonix regia* may probably be due to the presence of several bioactive anti-inflammatory principals. However, it needs isolation, structural elucidation, and screening of any of the above-mentioned active principle/s to pin point activity of drug. It is thus apparent that ethanol extract of *Delonix regia *possesses anti-inflammatory activity.

## Figures and Tables

**Table 1:** Effect of ethanol extract of *Delonix regia* (100, 200, and 400 mg/kg) on paw volume in carrageenan-induced paw edema rats.

Treatment	Dose mg kg^−1^	Carrageenan-induced rat paw edema Mean ± SEM*			
		+1 h	+3 h	+5 h	+7 h
Control	—	0.36 ± 0.06	0.79 ± 0.05	0.64 ± 0.04	0.68 ± 0.01
Indomethacin	10	0.18 ± 0.03^a^	0.27 ± 0.04 (65.82)^b^	0.29 ± 0.03 (54.68)^b^	0.33 ± 0.06 (51.47)^b^
EEDR	100	0.28 ± 0.02 (22.22)^NS^	0.46 ± 0.03 (41.77)^b^	0.39 ± 0.03 (39.06)^b^	0.41 ± 0.03 (35.93)^b^
EEDR	200	0.25 ± 0.01 (30.55)^NS^	0.43 ± 0.01 (45.56)^b^	0.38 ± 0.03 (40.62)^b^	0.39 ± 0.03 (42.64)^b^
EEDR	400	0.21 ± 0.02 (41.66)^NS^	0.41 ± 0.02 (48.10)^b^	0.37 ± 0.01 (42.18)^b^	0.4 ± 0.01 (41.17)^b^

*The number of animal was 6 in each group. Figure in parenthesis indicates percent inhibition in paw volume. The probability values were calculated using one way ANOVA followed by Dunnet's *t*-test: a < 0.05, b < 0.01, NS: not significant.

**Table 2:** Effect of ethanol extract of *Delonix regia* (100, 200 and 400 mg/kg) on cotton pellet granuloma in rats.

Treatment	Dose mg kg^−1^	Cotton pellet granuloma
		Weight of pellets Mean ± SEM
Control	0.3 mL	51.73 ± 2.35
Indomethacin	10	19.82 ± 2.52 (61.68)^b^
EEDR	100	37.24 ± 2.00 (27.99)^NS^
EEDR	200	30.85 ± 3.72 (40.36)^b^
EEDR	400	29.77 ± 3.76 (42.44)^a^

*The number of animal was 6 in each group. Figure in parenthesis indicate percent inhibition in cotton pellet granuloma. The probability values were calculated using one way ANOVA followed by Dunnet's *t*-test: a < 0.05, b < 0.01, NS: not significant.
